# Nano revolution: pioneering the future of water reclamation with micro-/nano-robots

**DOI:** 10.1039/d3na01106b

**Published:** 2024-03-05

**Authors:** Subham Preetam

**Affiliations:** a Department of Robotics and Mechatronics Engineering, Daegu Gyeongbuk Institute of Science and Technology Daegu-42988 South Korea sspritamrath93@gmail.com; b Institute of Advanced Materials, IAAM Gammalkilsvägen 18 Ulrika 59053 Sweden

## Abstract

Earth's freshwater reserves are alarmingly limited, with less than 1% readily available. Factors such as industrialisation, population expansion, and climate change are compounding the scarcity of clean water. In this context, self-driven, programmable micro- and nano-scale synthetic robots offer a potential solution for enhancing water monitoring and remediation. With the aid of these innovative robots, diffusion-limited reactions can be overcome, allowing for active engagement with target pollutants, such as heavy metals, dyes, nano- and micro-plastics, oils, pathogenic microorganisms, and persistent organic pollutants. Herein, we introduced and reviewed recent influential and advanced studies on micro-/nano-robots (MNR) carried out over the past decade. Typical works are categorized by propulsion modes, analyzing their advantages and drawbacks in detail and looking at specific applications. Moreover, this review provides a concise overview of the contemporary advancements and applications of micro-/nano-robots in water-cleaning applications.

## Abstract 2

Future of micro-/nano-robots in water reclamation.

## Graphical abstract

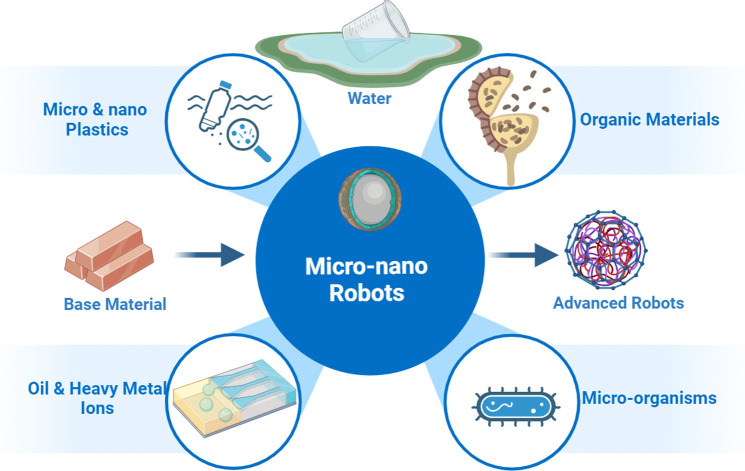

## 1. Introduction

Amid the growing environmental pollution issues, the emergence of micro- and nano-robot technologies has introduced innovative solutions for tackling and alleviating environmental pollutants.^1,2^ These advanced micro-/nano-robots are at the forefront of technology, enabling precise and targeted cleaning operations in complex environmental conditions.^3^ Due to their small size and ability to navigate complex structures, they offer excellent pollution removal with negligible ecological impact.^4,5^ Micro-/nano-robots are in line to attain sustainable conservation by eliminating the need for conventional, frequently dangerous cleaning methods.^6,7^ MNRs have recently emerged as a compelling technology that provides the opportunity to actively maneuver micro-/nano-particles in an aqueous medium while promising high interaction efficacy and spatiotemporal precision.^8^ The development of MNRs has enabled a variety of micromachines that can be driven by light,^9,10^ magnetic field,^11–13^ or chemical^14–17^ methods for pollutant removal and degradation.

**Fig. 1:**
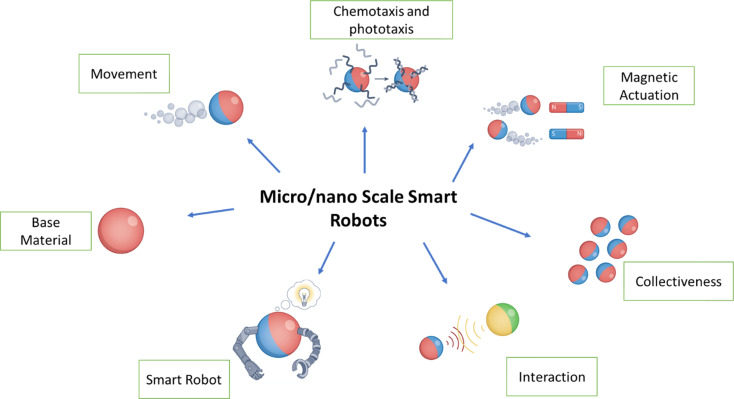
Micro- and nano-robots constructed using micro- and nano-scale materials.^16^

Important aspects that have been explored encompass their abilities for movement, versatility, adaptive reactions, collective behaviour, and interactions among micro-/nano-robots.^18,19^ This review also explores various techniques for the breakdown and removal of pollutants and highlights key developments in water treatment.^1,20–22^ It also highlights the essential elements to consider while building these systems to increase their efficiency in dealing with various pollutants.^23^ These robotic systems showcase adaptability when addressing various environmental cleaning issues.^24–26^ They can handle tasks ranging from managing oil spills in water ecosystems to purifying water and air sources.^19,27^ Many of these micro-/nano-robots are capable of autonomous operation, which lessens the need for human involvement and allows for real-time monitoring and data collection during cleaning procedures, as depicted in Fig. 1.

Micro-/nano-robots hold great potential for transforming environmental cleaning, underlining the importance of collaborative efforts across various fields, including robotics, materials science, environmental science, and engineering.^28–30^ While substantial advancements have been achieved, there are still hurdles to overcome, such as scalability, cost-effectiveness, and ensuring compatibility with natural ecosystems.^31^ Furthermore, contemporary water-remediation techniques often need to be revised as they either fail to eliminate pollutants or produce harmful byproducts that may harm the environment. To fully unlock the capabilities of these technologies, these challenges must be addressed. In conclusion, this review explores the existing obstacles and outlines future pathways, offering insights into the practical applications of intelligent micro- and nanorobots in tackling urgent water quality concerns.^26,32^

**Fig. 2:**
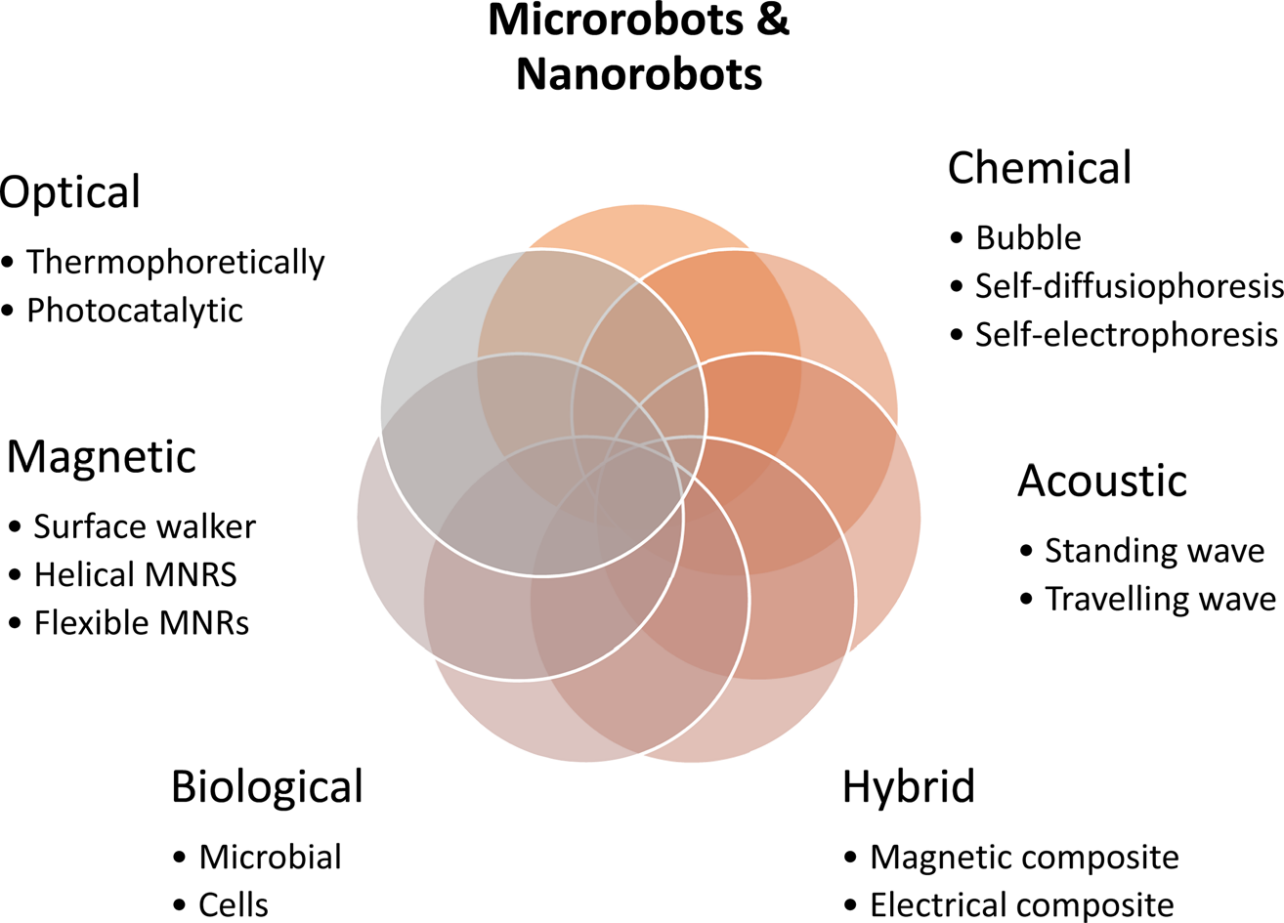
Different types of MNR on the micrometer or nanometer scale that can be controlled through proper actuation to accomplish specific tasks.^1^

In Fig. 1, it can be seen that materials produced at the micro- and nanoscales that are used to build MNRs tend to possess several essential characteristics, including (i) propulsion: these robots can move independently, either by using chemical fuel or in response to external forces;^33,34^ (ii) multifunctionality: they are versatile and capable of performing specific tasks, making them adaptable for various applications;^35,36^ (iii) taxis: MNRs can adapt to environmental cues, such as exposure to light (phototaxis), gradients in chemical substances (chemotaxis), or magnetic fields (magnetotaxis);^7,37^ (iv) communication: they can communicate with nearby robots, enabling synchronized operations while exchanging vital information;^32^ (v) collective behavior: the robots can collaborate and work together in groups, enhancing process efficiency or undertaking complex tasks beyond the capabilities of individual units.^32^ MNRs typically range from 10 nm to 100 μm, while nanorobots are smaller, measuring less than 1 μm.^8,16,24,28,38^ For example, the ideal magnetic MNR would have a magnetic engine at its heart, allowing it to precisely regulate its position, speed, and trajectory by adjusting the parameters of the magnetic field, as shown in Fig. 2.^6,39^ To maximize the effectiveness of adsorption, the core engine is usually encased in an adsorber with a large portion of surface area, which is often attained by using very porous materials. Covering this adsorption layer with a shell constructed of a photosensitive substance that can undergo multiple photodegradation activities is also possible.^2,40,41^ To improve the breakdown of contaminants and increase the effectiveness of degradation, the robots' surfaces may also be changed with enzymes.^42^ Evaluation of the stability of enzymes in the presence of light and photogenerated reactive oxygen species (ROS) is essential.^43^

This article provides an overview of recent advancements in the field of microrobots and nanorobots (MNRs), as illustrated in Fig. 2. The categorization of MNRs based on their driving mechanisms is a central focus, with the classification encompassing magnetic, optical, chemical, biological, and hybrid actuation. The examination of the propulsion methods includes electrical fields and ultrasound. While conventional techniques have significantly contributed to the evolution of MNRs, their limited compatibility with biological (especially in environmental or biomedical^44–50^) applications restricts their potential.^14,15,32,35,51^ Within each category, the review delves into the mechanisms of the considered MNRs, outlining their respective advantages, disadvantages, and highlighting noteworthy contemporary research toward better water reclamation.^52^ The article culminates with a comprehensive summary, analysis, and discourse on the existing challenges, concluding with the prospects for future developments in MNRs in this evolving field of water reclamation.

## 2. Benefits of MNRs as a booming field for the future environment

• Cutting-edge technology: MNRs represent the forefront of technological innovation in environmental remediation. These miniature machines offer a novel approach to addressing various forms of environmental pollution by employing advanced engineering principles and materials science.

• Precision cleaning: MNRs possess a remarkable ability to navigate complex environments with unparalleled precision. Their small size and maneuverability enable the targeted and effective cleaning of contaminated areas, minimizing collateral damage to the surrounding ecosystems.

• Minimizing environmental impact: one of the key advantages of MNRs is their potential to reduce the use of harmful chemicals in cleaning processes. By utilizing these robots, we can minimize disruption to ecosystems and mitigate the adverse effects associated with traditional cleanup methods.

• Versatility: micro-/nano-robots can be designed and tailored for a wide range of environmental cleaning tasks. From cleaning up ocean oil spills to removing pollutants from water sources and air, MNRs offer versatile solutions to diverse environmental challenges.

• Autonomous operation: many micro-/nano-robots are equipped with autonomous capabilities, reducing the need for constant human intervention and monitoring. This autonomy enables them to operate efficiently and effectively in remote or hazardous environments.

• Sustainability: the small size and energy-efficient designs of MNRs align with sustainability goals. Their reduced environmental footprint and ability to perform tasks with minimal energy consumption make them a promising solution for long-term environmental cleanup efforts.

• Real-time monitoring: some micro-/nano-robots are equipped with sensors that provide real-time data and feedback during cleaning operations. This capability allows for the continuous monitoring of pollution levels and the effectiveness of cleanup efforts, enabling adaptive strategies for environmental remediation.

• Interdisciplinary collaboration: success in the field of micro/nanorobotics for environmental cleanup often requires collaboration between various disciplines, including robotics, materials science, environmental science, and engineering. Interdisciplinary collaboration fosters innovation and enhances the development of effective MNR-based solutions.

• A greener future: the application of micro-/nano-robots in environmental cleaning holds promise for a cleaner and more sustainable future. By mitigating pollution and preserving ecosystems, MNRs contribute to efforts aimed at creating a healthier environment for current and future generations.

## 3. Applications in water treatment

### 3.1 Removal of nano- and microplastics

The building blocks of plastics are polymer chains created by joining monomers with covalent connections. Since the 1950s, there has been considerable growth in the production of plastics due to the widespread usage of plastics and their inherent adaptability, resilience, adaptability, lightweight, and affordability. The problem with plastics is that they tend to concentrate in marine habitats, where they slowly degrade into smaller, more dangerous particles known as microplastics (particles smaller than 5 μm) and nano plastics (particles smaller than 1 μm), which are difficult to eliminate.^43,44,53^ The most often used ones include polypropylene, polyethene, and polystyrene. Nano and microplastics can attract contaminants to their surfaces and help bacteria build biofilms because of their unique physical and chemical properties.^14,32,43,54^ These minute plastic particles risk the well-being of all living things because they can pollute water supply systems or invade the food chain.

Initial efforts to employ micro-nanorobots for capturing microplastics involved using light-powered Au–Ni–TiO_2_ microrobots, which are self-sufficient microscale machines.^16,53^ When these microrobots are subjected to ultraviolet (UV) light, they generate oxygen radicals and heat, permitting them to catch and eradicate microplastics from water. Besides, magnetic nanorobots have been designed and manufactured with Ni–TiO_2_ catalyst layers and magnetic metals to transport to microplastics. Upon UV-light illumination, these nanorobots become powered by catalysis and enable efficient microplastic removal. Their small size allows them to access confined environments, and they provide a sustainable method for pollution mitigation in water systems.

On top of this, they are environmentally friendly because they have a reduced energy requirement and no chemical byproducts, which avoids secondary contamination. Robust adhesion to microplastics is facilitated through the efficient chemical modifications of the Ni–TiO_2_ catalyst surface and magnetic metallic elements. This attribute is vital for the efficient and selective separation of these plastics from water, even when competing for removal. Ultimately, micro- and nanorobots present a promising approach for capturing and eradicating microplastics in water systems, safeguarding the ecosystems from their damaging consequences.^22,55^ Despite the progress in using micro- and nanorobots to target and remove microplastics from water, some limitations and challenges remain to be addressed. For instance, achieving the efficient and economical large-scale deployment of these robots for environmental cleanup is still a work in progress. Furthermore, ensuring that these technologies do not inadvertently harm natural ecosystems or organisms is a critical concern that requires careful consideration and research. Nonetheless, the use of micro- and nanorobots to combat microplastic pollution in water is a promising avenue for addressing this growing environmental challenge.^14,54^

Similarly, magnetic microrobots designed after pollen grains from sunflowers can efficiently remove large amounts of plastic at a low price.^22^ According to the applied magnetic field, these microrobots display a variety of motion modes: rolling, spinning, and wobbling. By adjusting the shaking motion, these ‘micro submarines’ can work together to gather, transport, and emit a bigger polystyrene bead (100 μm) or create strands to move tiny beads of polystyrene out due to the flow of fluid produced by their motion.^22^

A further strategy for eliminating microplastics is the use of an adsorptive bubble separation process. This method uses microrobots with a core and shell made of hydrothermally synthesized Fe_2_O_3_–MnO_2_.^55^ As these microrobots move, they generate O_2_ bubbles through the decomposition of H_2_O_2_. These oxygen bubbles entrap microplastics, and the bubbles and microplastics propel themselves closer to the solution's surface.^55^ This method produces foam that is simple to separate. The benefit of this microrobot is that it can navigate by using asymmetric illumination rather than costly metal coatings, which is remarkable.

**Fig. 3:**
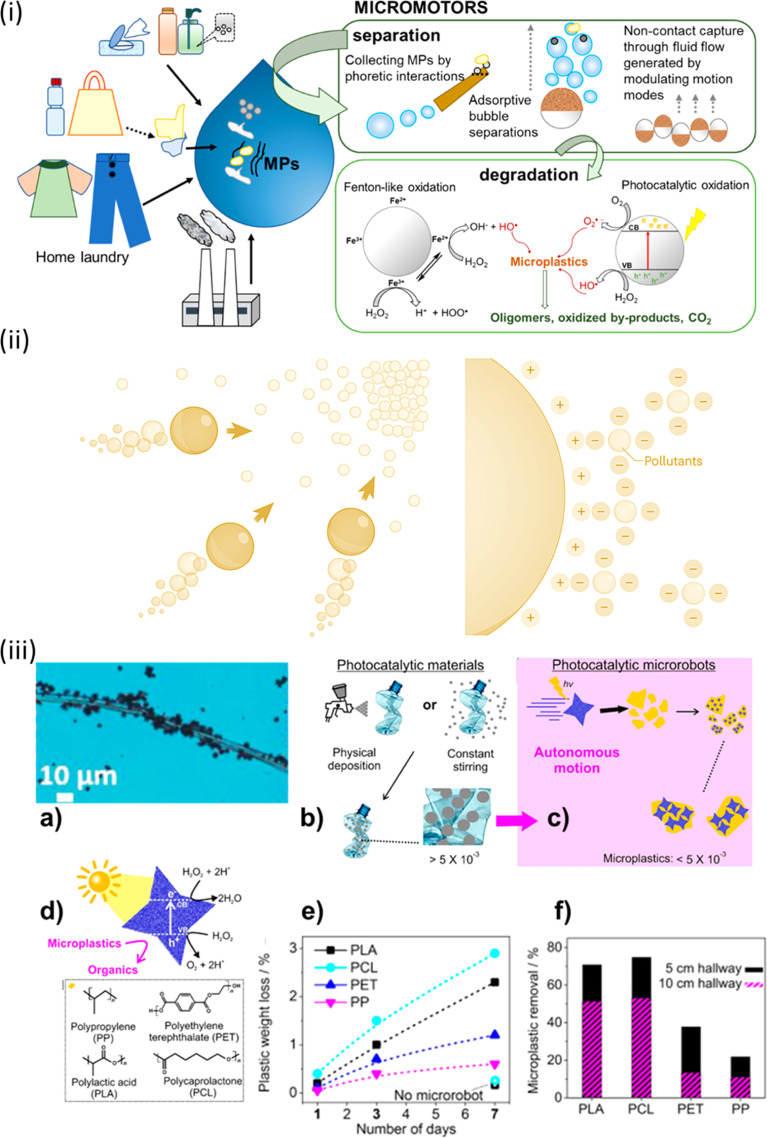
(i) Micromotors as efficient tools for microplastic separation and degradation. (ii) Bubbles direct micro-/nano-robots in the direction of areas that have greater contaminant concentrations. They can electrostatically absorb pollutants with an opposing surface charge. (iii) Light-driven microrobots demonstrate the photocatalytic degradation of microplastics in this study. In (a) microrobots could be observed swarming on a polymer textile fiber, actively participating in the degradation process. In contrast, (b) illustrates conventional methods that maximize contact between BiVO_4_ microrobots and waste, employing physical deposition and constant stirring techniques. (c) Autonomy of BiVO_4_ microrobots, where their independent motion under sunlight allows them to attach to floating microplastics, (d) the self-propulsion of BiVO_4_ microrobots alongside the polymeric microplastic waste under investigation. (e) Efficacy of the treatment, depicting the weight loss of microplastics after 1, 3, and 7 days of exposure to microrobots in a 7 mL aqueous solution containing 0.01 wt% H_2_O_2_. (f) Efficiency of microplastic collection, including various types, such as PLA, PCL, PET, and PP, in channels of different sizes (5 cm in black and 10 cm in magenta). Reprinted from ref. 56. Copyright 2022, American Chemical Society.

To remove and degrade microplastics in narrow spaces, Fe_3_O_4_–BiVO_4_-based microrobots have been used. These devices are driven by light and magnetism. Due to their intrinsic asymmetry, these microrobots move when exposed to visible light and are composed of star-shaped microparticles, as shown in Fig. 3.^16,56^ They move when there is 0.1% H_2_O_2_ present. They can securely adhere to substantial plastic objects made of polylactic acid, polycaprolactone (PCL), polyethene terephthalate (PET), and polypropylene. The microplastics' surface properties and morphology degrade after exposure to visible light and H_2_O_2_ for a week. However, there is only a slight weight loss (3% for polylactic acid) and the breakdown efficiency is still poor. To improve the enzymatic breakdown of microplastics, lipase was selectively added to Fe_3_O_4_ microrobots coated in polydopamine (PDA). Due to the bonding qualities of PDA, swarms of these microrobots could stick to and transfer bigger microplastic particles (up to 140 m) when activated by a transverse spinning magnetic field. After incubation with the microrobots for a whole night, optical microscopy proved that enzymes had broken down the PCL microplastics.^16,56^ These systems can move and retrieve these plastic parts from an intricate structure of bigger channels with surprising efficiency due to their attachment.

### 3.2 Removal of organic molecules

The ability of numerous micro- and nanorobots intended for the purification of water to eliminate or deteriorate pollutant substances, like methylene blue and rhodamine B, which are frequently used as dyes, was evaluated.^21^ Further, these robots have also shown potential in combating more resilient and dangerous pollutants, such as hormones, antibiotics, chemical warfare agents, phenolic substances, nitroaromatic compounds, and psychotropic drugs. Although these micro- and nanorobots have great potential, issues with their production costs, operational costs, and performance indicators, like efficiency and reusability, limit their broad use in practical applications.^21^

Biohybrid microscopic robotic devices can solve the problems caused by hazardous fuels and costly noble metal catalysts, and integrate biological microorganisms with synthetic components. For instance, the pesticide methyl paraoxon, a simulated nerve agent, which is a very stable substance that is difficult to degrade due to its chemical solid interactions, was successfully eliminated using microrobots driven by marine rotifers, microorganisms common in aquatic settings.^57^ The rotifers were filled with microbeads carrying organophosphorus hydrolase (OPH) in this procedure. The microbeads were surrounded by polluted water propelled by the cilia in the mouth of the rotifers. As a result, methyl paraoxon hydrolyzed faster and became the electrochemically traceable *p*-nitrophenol.^21,57^

Consequently, the deterioration rate was much improved, increasing eightfold compared to simply employing microbeads with OPH functionality. Using polypyrrole–Fe_3_O_4_–Pt tubular microrobots is an innovative strategy for eliminating the synthetic hormone α-estradiol.^35^ The polypyrrole surface of these microrobots may be altered by modifying the pH of the solution to increase their magnetism to the hormone. They also featured an inner Pt layer for bubble propulsion in H_2_O_2_, Fe_3_O_4_ nano-particles for magnetic steering, and Fe_3_O_4_ nano-particles for magnetic steering.^35,58^ Surprisingly, when α-estradiol was added to a solution, the hormone stuck to the moving microrobots and formed thick fibers that resembled spiderwebs. Then, utilizing an external magnetic field, these microscopic robotic devices and the woven α-estradiol webs could be combined into a single, portable unit that could be effortlessly detached from the treated water.

Micro- and nanorobots play a vital role in environmental remediation by enhancing fluid mixing, and accelerating the removal or degradation of pollutants. While higher robot speeds may intuitively improve efficiency, a counterintuitive example involves Pt-hematite Janus microrobots with thicker Pt coatings, which showed increased speed but also led to faster H_2_O_2_ intake, reducing the degradation efficiency. In contrast, a ‘microrobots in sponge’ approach used microrobots made of cobalt ferrite incorporated into a porous polyurethane sponge. This setup could capture more pollutants, allowing the microrobots to conduct efficient *in situ* degradation through the Fenton reaction with minimal H_2_O_2_.^59^ As a result of this synergy, methylene blue could be rapidly degraded in large quantities, recovered, and then used again by microrobots. Utilizing laccase is a method for enzymatically eliminating organic compounds from water.^60^

**Fig. 4:**
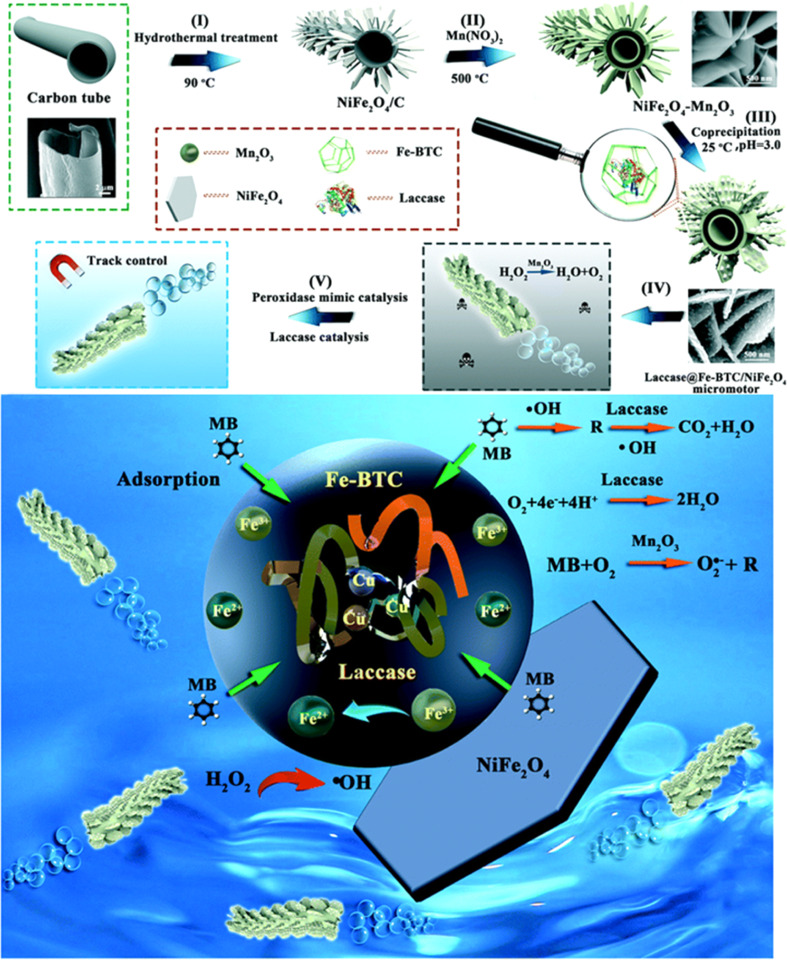
Schematic representation detailing the preparation and utilization of the laccase@Fe-BTC–NiFe_2_O_4_ nanozyme hybrid micromotor. (I–V) The process begins with the synthesis of carbon tubes using kapok as a template, followed by using NiFe_2_O_4_ and laccase-immobilized Fe-MOFs to form NiFe_2_O_4_/Mn_2_O_3_. Finally, the degradation of organic compounds is facilitated by combined natural-enzyme and nanozyme catalysis processes. Reprinted from ref. 60. Copyright 2020, RSC.

Yang *et al.* presented a micromotor that had a unique hierarchical structure, where laccase-immobilized-Fe-BTC MOF nano-particles grew uniformly on Mn_2_O_3_–NiFe_2_O_4_ nanosheets, which was constructed using natural kapok fiber as a template, as shown in Fig. 4. This micromotor was capable of self-propulsion with oxygen bubbles *via* the decomposition of H_2_O_2_ catalyzed by Mn_2_O_3_.^60^ While laccase tends to lose its enzymatic activity when subjected to UV light, attaching photosensitive azobenzene substances to microrobots assists in safeguarding the enzymes, like horseradish peroxidase and catalase. When subjected to direct UV-light irradiation, this protection permitted the enzymatic breakdown of diverse substrates, and it exhibited much higher catalytic activity to methylene blue degradation than their passive counterparts.

To locate and remove the most dangerous contaminants from wastewater, micro- and nanorobots must display selective behavior.^60^ MIPs, or molecularly imprinted polymers, present a potential solution to this problem. In this method, a substance (called the matrix) is created by imprinting a molecule (also known as the template) onto it. For instance, MIP-mediated selective recognition can be made possible by imprinting the template, *i.e.*, the antibiotic erythromycin, onto a matrix known as a thermoresponsive poly(*N*-isopropylacrylamide) (PNIPAM) hydrogel coating of Mn_3_O_4_–CoFe_2_O_4_ microrobots that were made utilizing lotus pollen as a permeable bio-template.^60^ This method enabled erythromycin to be adsorbed and released under temperature control.

A major drawback of MNRs is the small navigational range, usually only a few millimeters, of micro- and nanorobots in water purification. This constraint becomes particularly challenging when they must function in enormous amounts of water, often measured in cubic meters. Researchers have consequently investigated the idea of self-powered “aircraft-like” carriers for photocatalytic microrobots to overcome this restriction. For instance, a millimeter-scale robot that was 3D printed and had a conical head and a tubular framework packed with Pt–TiO_2_ microrobots and ethanol was reported that can travel over tens of meters.^16,47,49^ The Marangoni effect, created by releasing microrobots and ethanol fuel asymmetrically and concurrently, causes movement. With this method, photocatalytic microrobots may be released gradually and across a wide area, enabling the breakdown of compounds like picric acid. While micro- and nanorobots have been extensively studied in deionized water for water purification applications,^40^ their effectiveness can be hampered by solid impurities commonly found in sewage samples. Future research should focus on testing these robots in real-world, contaminated specimens to assess their practical viability.

### 3.3 Heavy metals separation

The presence of heavy metals is a severe environmental problem, especially given the persistent and long-lasting nature of heavy metals, including arsenic, mercury, cadmium, lead, and copper, which are significant pollutants of water.^61^ These metals build up in living things and can cause significant health problems by producing reactive oxygen species (ROS) that cause oxidative damage. A possible method for removing heavy metals is to use self-propelled micro- and nanorobots.^30^

Micro- and nanorobots may be produced for a much more affordable price if their essential parts are made of easily accessible and natural materials. For instance, the lattice mismatch that caused halloysite nano clay to emerge over millions of years by rolling kaolin clay into small tubes makes it a superb absorber.^62^ Nanoclay robots can effectively eliminate Zn^2+^ and Cd^2+^ when coated with Pt by producing H_2_O_2_ bubbles and by electrostatically attracting metal ions.^63^ Another helpful substance is pollen grains, which are renowned for their durability and biocompatibility. Pt-coated pollen grains exhibit exceptional Hg^2+^ elimination performance. Microrobots have also been created using kapok fibers and spirulina, an edible alga with a helical structure, for a variety of pollution-removal tasks.^16,64^

**Fig. 5:**
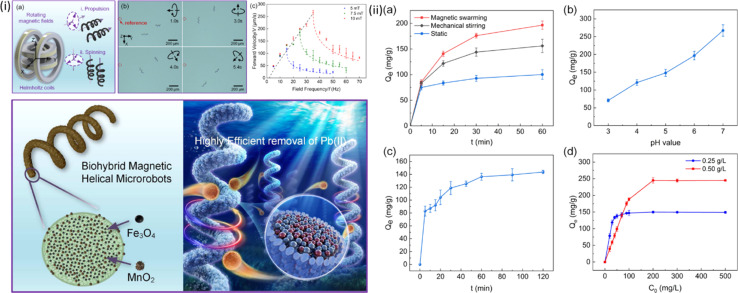
(i(a)) Schematics of a magnetic nanorobot (MNR) demonstrating cork-screw propulsion and vertical spinning under magnetic actuation; (b) time-lapse images capturing the MNR in action, propelled directionally by a vertical rotating magnetic field; (c) forward velocity–frequency plots under varying strengths of vertical rotating magnetic fields; (ii(a)) adsorption performance of the MNR in diverse working conditions, including the static state, mechanical stirring, and magnetically actuated spinning in swarms, (b) impact of the initial solution pH value on the adsorption performance, (c) influence of the processing time on Pb(ii) adsorption; (d) effect of the initial Pb(ii) concentrations on the overall Pb(ii) adsorption. Reprinted with permission from ref. 51. Copyright 2021, American Chemical Society.

One example is the production of magnetic microrobot swarms capable of extracting Pb^2+^ from water without needing H_2_O_2_ when Fe_3_O_4_ and MnO_2_ nano-particles are grown on spirulina, as shown in Fig. 5.^51^ An idea for metal-free C_3_N_4_ tubular microrobots powered by visible-light irradiation has been put forth to lessen the dependency on costly Pt engines.^29^ Here, H_2_O_2_ is broken down by the semiconductor's photogenerated carriers, creating bubbles that aid in self-propulsion. By making complexes with functional groups based on N and C, these microrobots can remove Cu^2+^. Surprisingly, the attached metal ions exhibit a Fenton-like behavior, speeding up the breakdown of H_2_O_2_ and increasing the rate of the microrobots. Additionally, this method may be used to collect priceless metal ions, like Pd^2+^ and Ag^+^.^65^

Increasing the adsorption capacity of micro- and nanorobots is not simple but is a highly effective method for improving the effectiveness of remediation. Metal–organic frameworks (MOFs) are compelling materials due to their extensive surface area, adjustable sizes of pores, and the organic linker's functional properties, which can be tailored to specific applications.^66^ For instance, superstructures made of asymmetric hollow silica nanobottles with an extensive surface area of about 600 m^2^ g^−1^, coated with Fe_3_O_4_ nano-particles, show self-propulsion in H_2_O_2_, allowing for the quick removal of Cu^2+^ (achieving an 80% removal rate in just 1 h).

Additionally, these MOF-based microrobots have exhibited efficiency in removing radioactive UO_2_^2+^ and other metal ions from water. Making robots that can target pollutants specifically is another way to boost the efficiency of micro- and nanorobots. In the case of polyaspartic acid and Cd^2+^, coordination effects and electrostatic interactions between amino and carboxyl groups cause this selectivity. Still, in the case of methylmercury, more substantial connections are created between the sulfhydryl groups in polycysteine and Hg. Similarly, the use of minerals known for their inherent affinity for certain heavy metals, such as illite and zeolite for the radionuclide Cs^+^, can create robots that can absorb substances selectively.

The electro absorption of heavy metals has been chosen using tubular microrobots composed of graphite nanofiber–Ni–Pt or Bi–Ni–Pt.^16^ The supercapacitor's system for storing charges inspired these robots. When these microrobots collide with a negatively charged electrode, electrons are transferred to them, enabling the electrostatic adsorption of metal cations in an O_2_-free solution. The layering of graphite and Bi is included in this adsorption, which is significant for reaching up to 400 layers from the surface of the microrobots and having the ability to incorporate metal cations in the interlayer gap. In this method, bigger cations, like Na^+^ and Ca^2+^, could be successfully removed by microrobots made of Bi, whereas graphite-based microrobots could capture Li^+^.^67^ The cations may be quickly released by moving the microrobots magnetically to an O^2^-rich solution, enabling various applications.

### 3.4 Cleaning oil spills by MNR

**Fig. 6:**
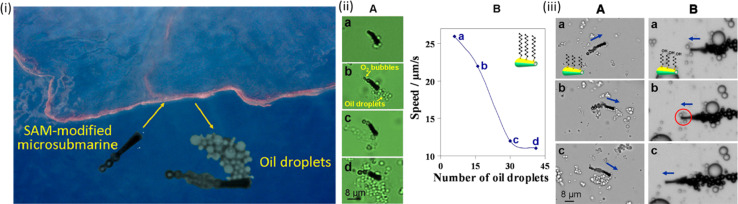
SAM-MNR carrying floating olive oil droplets. (i(A)) Images (a–d) were taken from video 2 after navigating in the water–oil interface. (B) Dependence of MNR speed upon the number of cargos (olive oil droplets); (iii) MNRs with different head functional groups interacting with tiny olive oil droplets. A hexanethiol-modified MNR can confine a payload of multiple oil droplets. (A and B) Arrows indicate the direction of MNR movement. Reprinted with permission from ref. 25. Copyright 2012, American Chemical Society.

More than one million metric tons of oil are released into the environment yearly due to the rise in tanker operations and subsequent oil spills into water bodies, which pose a severe threat to the ecosystem and demand quick and efficient remedies. A self-organized monolayer of extensive alkanethiol chains was added to Au–Ni–poly(3,4-ethylenedioxythiophene) (PEDOT)–Pt microtubes^25^ as an early example of employing micro- and nanorobots for oil cleaning. Because of their powerful interactions with hydrophobic oil droplets, these microrobots could collect and transfer oil spills in H_2_O_2_. To reduce the costs and toxicity while sacrificing some of their lifespans, Pt and H_2_O_2_ were replaced with a disintegrating engine, as shown in Fig. 6.^25^

New methods for creating hydrophobic microrobots have been proposed. One approach involved modifying microfluidic double emulsions with Fe_3_O_4_–Ag nano-particles, resulting in porous polymeric spheres that can powerfully propel the robots in H_2_O_2_ while capturing oil and being reusable.^68^ Another method used electrospinning to create walnut-like microrobots using PCL, Fe_3_O_4_ nano-particles, and catalase, enabling bubble propulsion in H_2_O_2_ with oil adsorption due to their hydrophobic surface and a photothermal effect under light.^69^

Due to their practical and ecologically acceptable method of operation, magnetically driven micro- and nanorobots have proven beneficial in removing oil. When a polyurethane sponge is modified with PDA and Fe_3_O_4_ nano-particles, the sponge's water absorption ability may be switched to superhydrophobic, creating a bifunctional platform for oil collection.^2,25^ Moreover, magnetic microsubmarines using sunflower pollen grains act under a similar process where the movement occurs at interfaces, such as water–oil and liquid–solid interfaces, enabling them to effectively adsorb and transport oil droplets much more significantly than their size might otherwise allow.^54^

To address incomplete oil spill degradation, enzyme-modified nanorobots inspired by biology have been proposed. For instance, mesoporous silica nano-particles adorned with *Candida rugosa* lipase, fueled by triacetin, break down soluble and slightly soluble triglyceride substrates (representing oil pollutants).^70^ Yolk@spiky-shell-structured nanorobots with lipase modification and near-infrared light responsiveness enabled precise oil droplet targeting, achieving a high degradation efficiency of approximately 90% within 20 min through a synergy of enzymatic and photothermal processes.^33^

### 3.5 Microorganisms' removal by MNRs

Pathogenic microorganisms, including bacteria, fungus, viruses, and protozoa, are the source of waterborne biological pollution.^4^ These pathogens pose significant threats to humans and animals, leading to widespread illness and death. Microorganisms can adapt to their environment, enter a dormant state for prolonged survival without nutrients, and resume growth when the conditions improve.^8,19,23^ They can also develop resistance to purifying techniques and create toxins as byproducts, which makes their complete eradication difficult. Disinfection methods rely on ozone, chlorine, chloramines, or UV therapy.^71^ However, these techniques involve energy-intensive operations (such as UV treatment), produce hazardous byproducts, and demand high disinfectant dosages to prevent the return of infections, which can develop hardy microbial biofilms. Additionally, in order to prevent future outbreaks, it is necessary to determine if standard disinfection techniques are effective against newly discovered fungal, bacterial, and viral strains.^72^

Initial efforts to eliminate aquatic pathogens were primarily aimed at exterminating bacteria in motion by enhancing microrobots with antibacterial substances, like antibiotics, enzymes, or protein receptors.^73^ However, because bacterial biofilms frequently grow in difficult-to-reach places, it is necessary to be able to direct the movement of the robots, which is commonly accomplished using external magnetic fields.^20^ Catalytic antimicrobial robots (CARs) equipped with iron oxide nano-particles have been developed to remove bacterial biofilms, resembling clogging plaque, in situations like the growth at the end of conical tubings.^74^

**Fig. 7:**
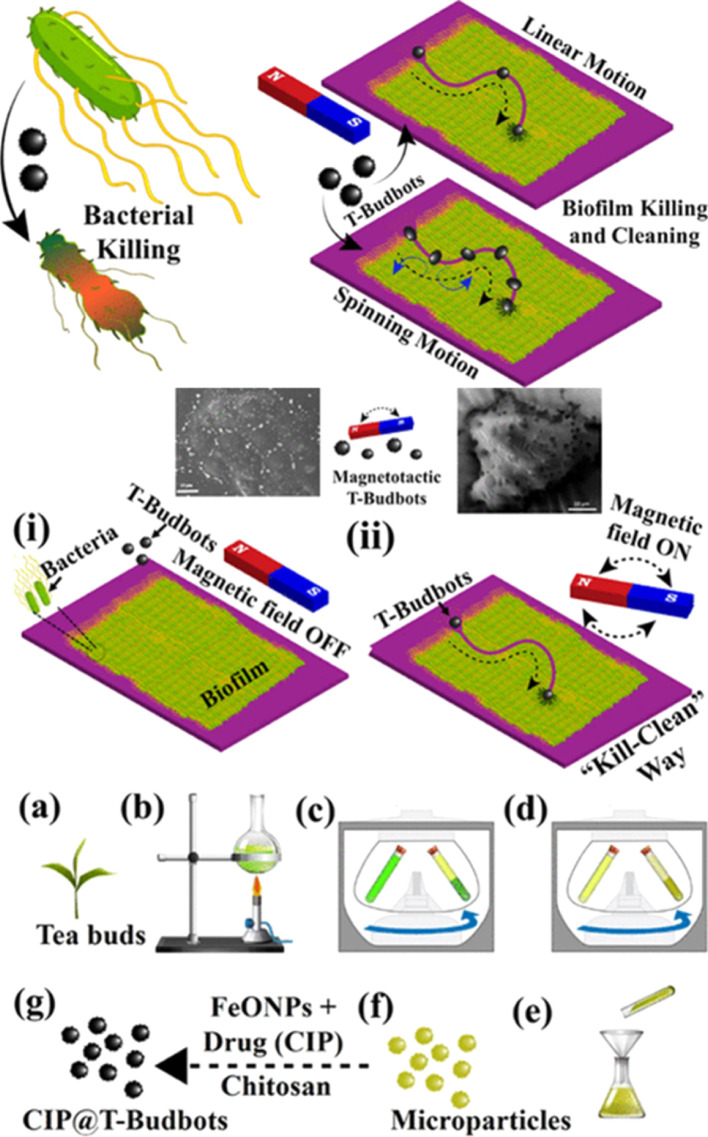
Schematic representation of (i) an intact bacterial biofilm grown on a substrate before exposure to T-Budbots, and (ii) the movement of magnetically driven T-Budbots across the biofilm surface, guided by a magnetic field, as part of a kill-n-clean approach for biofilm disruption and removal. Additionally, the schematics outline the process of collecting tea buds, preparing tea extract, and subjecting it to differential centrifugation at varying speeds, followed by filtration to produce microparticles ranging in size from 50 to 160 μm. Subsequently, these microparticles were decorated with chitosan-modified FeONPs and loaded with the drug CIP to create CIP@T-Budbots. Reprinted from ref. 75. Copyright 2020, American Chemical Society.

It is possible to drill into and remove biofilm matrices using helicoidal CARs when they are exposed to H_2_O_2_, mutanase–dextranase enzyme solution, and a rotating magnetic field (5 μm s^−1^). In addition, the catalytic activity of CARs clears away any leftover bacteria, preventing regrowth and thoroughly removing rigid bacterial biofilms.^75^ The “kill-n-clean” strategy, a distinct method, employs magnetic microrobots constructed from biodegradable permeable tea buds. These microrobots containing ciprofloxacin covered with Fe_3_O_4_ nano-particles use the acidic microenvironment inside the bacteria biofilms to produce antibiotics in a pH-dependent manner that leads to biofilm removal and breakdown,^75^ as shown in Fig. 7. Furthermore, magnetically triggered helical microswimmers with an inner carbon core combine light and magnetic fields to create a photothermal reaction based on near-infrared light, effectively killing *E. coli* bacteria. Micro- and nanorobots driven by light eradicate bacterial biofilms by combining autonomous motion with ROS generation, causing significant biofilm destruction and hindering regeneration.^74^ For instance, naturally asymmetric Ag-doped ZnO microrobots could move independently under UV light and exhibited increased efficacy in the eradication of *Pseudomonas aeruginosa* and methicillin-resistant *Staphylococcus aureus* (MRSA) biofilms (approximately 50% eradication in 5 min compared to 10% without light).^17^ Alternatively, Ag-coated TiO_2_ nanotube nanorobots efficiently eliminated bacterial biofilms of multiple kinds adhered to metal surfaces (40% eradication in 30 min) with versatile motion patterns under UV to visible light. When exposed to visible light, star-shaped BiVO_4_ microrobots produced chemical gradients that assisted them to move around, group together into swarms, and capture and destroy aquatic fungal microorganisms, like *Saccharomyces cerevisiae*.^76^

Biohybrid robots are an exciting option for large-scale disease control. For instance, self-driven *Chlamydomonas reinhardtii* microalgae, chemically linked to the angiotensin-converting enzyme 2 (ACE2) receptor using click chemistry, could effectively eliminate the SARS-CoV-2 viral spike protein (95%) and pseudo virus (89%).^77^ These biohybrid microbots can operate without additional fuel in various water environments, such as acetate phosphate medium, phosphate-buffered saline, drinking water, and river water, for up to 24 h at a time and for up to five consecutive cycles.

## 4. Challenges and future directions of MNRs

Tiny robots are like superheroes for cleaning water. These micro- and nanorobots are super small and can move independently, helping to get rid of pollutants. They are especially good at cleaning water, but using them in big open areas like oceans is challenging. Some robots can be powered by sunlight, but they may not work well in deep water or high-salinity areas. To make them even more powerful, scientists are combining different ways for them to move and clean. While these tiny robots show great promise, making them simple, cost-effective, and environmentally friendly is crucial for them to be widely used. The goal is to create efficient, safe, and affordable solutions for cleaning our environment with these fantastic micro- and nanorobots.

Micro- and nanorobots do not seem appropriate for extensive open-water body restoration, including oceans. However, they may be used successfully to remove or degrade toxins in small areas, such as when integrated into a ship for offshore activities. A tank with inlet and output pipes for polluted and cleaned water should be part of a small robot-based water treatment system. Sensors should be included in the system to monitor the quality of the treated water. If required, these robots can run on UV-light sources or direct sunshine, with the help of H_2_O_2_ fuel. Particularly in salt water, where high conductivity can make it difficult for most micro- and nanorobots driven by light to move, magnetic activation could be selected over light-driven propulsion. For improved pollutant adsorption or degradation, the tank should be surrounded by several perpendicular magnetic coil pairs that an external controller can control. This will make it easier for the robots to move about. Additionally, it permits the outflow of cleaned water while collecting robots at a predefined spot after treatment.

Magnetic actuation makes robots' reusability possible, which may direct robots to a secondary vessel where non-biodegradable contaminants can be purposefully expelled. To satisfy these technological requirements, a perfect micro- or nanorobot would include various parts, each with a specialized purpose.

The system needs to be environmentally friendly and cost-effective compared to traditional water distribution systems. A comprehensive economic evaluation should include energy use for water pumping, water quality sensors, magnetic control systems, lighting, and treatment duration. These factors depend on the pollutant type, robot quantity, magnetic and light parameters, and removal efficiency.

MNRs have successfully addressed numerous technical challenges associated with various propulsion methods. Advances in the manufacturing process of untethered robots have significantly reduced their size to the nanometer scale. The incorporation of programmable paths and precise navigation has empowered microswimmers to access difficult-to-reach areas and deliver cargos to targeted locations with precision and autonomy. The introduction of soft MNRs further enhances their versatility, allowing them to navigate between obstacles without causing damage due to their flexible and pliable nature.

**Fig. 8:**
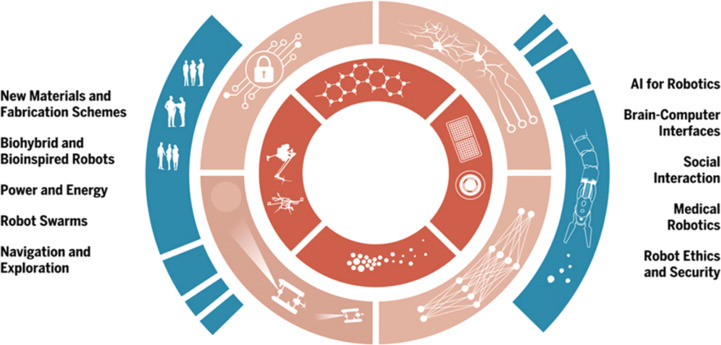
Main challenges in future robotics need to be resolved.^31^

Regarding the challenges, while the potential of micro-/nano-robots in environmental cleaning is wide, challenges like scalability, cost-effectiveness, and environmental compatibility must be addressed for their widespread adoption. The potential of micro-/nano-robots in environmental cleaning is vast and promising. These tiny robots offer innovative solutions for tasks such as pollutant removal, water purification, and environmental remediation. However, their widespread adoption faces several challenges that need to be addressed, as shown in Fig. 8.

One major challenge is scalability. While micro-/nano-robots have shown effectiveness on a small scale in laboratory settings, scaling up their production and deployment to tackle real-world environmental challenges is a significant hurdle. Developing manufacturing processes that can produce these robots in large quantities while maintaining quality and consistency is also essential for their practical use on a larger scale.

Cost-effectiveness is another critical factor that needs attention. Currently, the production and deployment of micro-/nano-robots can be costly, limiting their accessibility and feasibility for widespread adoption. Finding ways to reduce production costs without compromising the performance and reliability of these robots is essential for making them more economically viable for environmental cleaning applications.

Furthermore, ensuring the environmental compatibility of micro-/nano-robots is essential. It is thus crucial to assess the potential ecological impacts of deploying these robots in natural environments and to mitigate any adverse effects that they may have on ecosystems. Designing micro-/nano-robots with biodegradable materials or incorporating mechanisms for safe disposal after use can help minimize their environmental footprint. Addressing these challenges will be crucial for unlocking the full potential of micro-/nano-robots in environmental cleaning and realizing their benefits for sustainable development and environmental protection. Collaboration between researchers, engineers, policymakers, and industry stakeholders will be essential to overcome these hurdles and drive innovation in this field. This progression marks a notable milestone in the development of nanorobotic technology, expanding its capabilities for intricate tasks and applications in diverse environments.

## 5. Conclusion

In this context, our discussion has centered on the utilization of MNRs in water reclamation. These applications encompass a diverse array of challenges, including the remediation of diverse pollutants, such as microorganisms, oil spills, heavy metals, organic molecules, and, notably, nano and microplastics. By effectively targeting and mitigating these sources of pollution, MNRs offer a promising avenue for achieving a greener, more sustainable future.

One of the key strengths of MNRs lies in their ability to operate at the nanoscale, allowing them to access and interact with environmental contaminants at a level of precision previously unattainable. Their small size also enables them to navigate complex environments with ease, reaching areas that may be inaccessible to conventional cleanup methods. Additionally, MNRs can be designed with specific functionalities tailored to the task at hand, enhancing their effectiveness in addressing environmental challenges.

Basically, the use of MNRs in environmental cleaning with water reclamation holds the potential to revolutionize our approach to pollution remediation. By leveraging their flexibility, adaptability, robustness, and precision, we can develop innovative solutions that not only address current environmental issues but also pave the way for more sustainable practices in the future. Furthermore, the interdisciplinary nature of MNR research encourages collaboration between scientists, engineers, policymakers, and industry stakeholders, fostering innovation and driving progress in the field. Moreover, MNRs offer valuable contributions to society and hold considerable promise for diverse applications, particularly in the realm of environmental cleaning. As we continue to explore and harness the capabilities of this transformative technology, we move closer to realizing a cleaner, healthier planet for future generations.

## Author contributions

S. P. contributed writing – review & editing, writing – original draft, resources, methodology, data curation & editing, visualization, validation, supervision, methodology, investigation, and conceptualization of the manuscript.

## Conflicts of interest

Declared none.

## Supplementary Material
